# Notes and News

**Published:** 1895-01-12

**Authors:** 


### NOTES AND NEWS.

Collected and Communicated.

The Girdlers' Company have made a generous distribution of £535 10s. amongst the charities, of which the London hospitals have come in for a large share.

Professor Kitasto has received a golden cup, in honour of his discovery of the bacillus of the plague, from the inhabitants of Nagasaki.

The Sultan has recently conferred honours on several well-known medical men. M. Monod, Director of the Assistance Publique, Professor Graucher, M. Roux, and M. Pozzi have been made Grand Officers of the Medjidieh Order.

New Year honours have been conferred on Dr. J. Russell Reynolds, President of the Royal College of Physicians, and Mr. John Eric Erichsen, President of University College. Both have been created baronets. In both cases the distinction will meet with unanimous approval.

It would be well if the public, even when moved by an appeal made at their door on behalf of a charity, were to send their donations to the hospital authorities direct. At Exeter unscrupulous persons have been collecting apparently on behalf of the Devon and Exeter Hospital, and thus defrauding the institution and the poor of much needed help.

The Charity Commissioners do not appear to have stepped in too soon to inquire into the expenditure of the Almshouse known as the Donnington Hospital, near Newbury. The charity is one of those ancient foundations which has prospered and increased in value until, in the present instance, an income of £2,000 is available to provide for the twelve poor men according to the founder's intention. The pensioners are liberally maintained, it is true, at an expenditure of £763 per annum, but the large remaining income, it appears, has been absorbed in management and repairs. Provision for the aged is one of the questions of the day, and here, it would seem, is a case where proper distribution, not money, is wanting.

Whilst the United States' navy is about to establish a medical corps, in the army the corps has been reduced by 15 officers.

Steps are being taken in America to organise a hospital corps for the navy. It is felt that its establishment would be of great value.

Sir W. T. Lewis has generously offered £1,000 to endow a bed in an accident ward now proposed for the Merthyr Hospital.

We are glad to learn that the appeal to endow and extend the Sarah Acland Home for Nurses, as a memorial of Sir Henry Acland's long and valued services at Oxford, is being liberally and extensively responded to. The sum required is £10,000.

The question of a site for the proposed new infirmary for Newcastle has again come under consideration. The most favoured scheme provides that a site on the Leazes, consisting of eight acres, shall be granted for the new infirmary.

Not only country folk, but Londoners also, often lose much time in searching for hospitals they would visit. We are glad to note that the Great Ormond Street Hospital for Children has done its best to aid patients by affixing to their letters of admission a most clearly marked map, giving the site of hospital and its approaches extending over a considerable distance, and directions *viâ* 'bus or rail are also added to it.

The Lord Mayor has promised to preside at a meeting, on behalf of the North-Eastern Hospital for Children, at the Mansion House, on January 28th, at 3 p.m. This hospital is in urgent need of support. Its situation in poor but populous Hackney renders it liable to be overlooked by richer London, and it is to be hoped that the coming meeting will be the means of making its work and needs known, as the institution is highly deserving of support.

We have appealed for our deserving hospitals at home, and must not forget to remind our readers who may be journeying south through the great Suez Canal that a little English-founded hospital is waiting there to tend any who may be in need of its help on the voyage. The Lady Strangford Hospital at Suez is a veritable haven to sick travellers, and we hope that those who do not need its aid for themselves will nevertheless encourage it for the sake of the less fortunate ones.

Messrs. Adlard and Son have published a diphtheria chart more especially for the purpose of recording the events of the disease in relation to its treatment by anti-toxin. It really is a scheme for case taking, arranged in a tabular form, appended to an ordinary temperature chart, and it no doubt will be useful as encouraging the methodical observation of the various details suggested by it. We would, however, enter a protest against the encouragement here offered to the evil habit of entering notes on the back of the chart, a habit which leads to endless embarrassment in comparing and collating numerous cases. If it is thought necessary to provide extra space the chart should be printed on a folded sheet, which can ultimately be opened out so that all the facts shall be on one side of the paper.

